# What Sexual Orientation Change Efforts Change: Evidence From a United States Sample of 72 Exposed Men

**DOI:** 10.7759/cureus.68854

**Published:** 2024-09-07

**Authors:** Donald P Sullins

**Affiliations:** 1 Research, The Ruth Institute, Lake Charles, USA; 2 Sociology, Catholic University of America, Washington DC, USA

**Keywords:** ex-gay, religion, sexual orientation, sexual orientation change efforts, therapy

## Abstract

Background: Voluntary non-coercive therapeutic interventions for adults are collectively known as sexual orientation change efforts (SOCE). Research on SOCE has reported global or average sexual orientation change, but not in more detail. This study addresses two questions: Does change consist primarily of reduced homosexual orientation or increased heterosexual orientation? Does change consist primarily of change in behavior or change in attraction?

Method: A convenience sample of 72 men who had completed SOCE was examined for the decrease in homosexual attraction, increase in heterosexual attraction, and corresponding changes in sexual behavior among those experiencing a homosexual-to-heterosexual shift. All measures were self-reported.

Results: Participants reported extremely high religiosity. A third (32%, N=23) reported a shift from general homosexual attraction (score of 4-6) to general heterosexual attraction (score of 0-2) on the Kinsey scale, which ranges from "exclusively heterosexual" (0) to "exclusively homosexual" (6). Among these, effect sizes (Cohen's d) for sexual fantasy and desire for romantic intimacy were larger for homosexual reduction (fantasy: -2.3, desire: -1.5) than for heterosexual increase (fantasy: +1.8, desire: + 1.0). Effect sizes for sexual behaviors, measured by kissing and sex relations, dropped the largest possible amount for homosexual behavior (kissing: -1.0, sex: -1.5, both statistically significant at p<0.004) while there was little to no increase in heterosexual behavior (kissing: +0.36, sex +0.38, both statistically non-significant at p>0.13).

Conclusion: Change consisted primarily of reduced homosexual orientation and change in behavior was much stronger than change in attraction. SOCE supported conformity to strong religious norms against homosexual behavior, but not attractions, for this group of extremely religious men. Implications for understanding SOCE-related sexual orientation change are discussed.

## Introduction

Sexual orientation change efforts (SOCE), an acronym coined by the American Psychological Association (APA), is a blanket term for a wide variety of interventions and self-help practices that support the possibility of altering one or more of the dimensions of sexual orientation, i.e., sexual attraction, behavior, or identification. Pursued almost exclusively by unusually active participants in religions that proscribe homosexual behavior, they are primarily oriented toward helping such persons reconcile their nonheterosexual impulses or practices with a deeply felt religious identity that they experience as being more core to the self than the dimensions of sexual orientation. Although often confused or conflated with “conversion therapy,” these practices are quite distinct: they do not seek to convert sexual orientation and do not usually consist of professional therapy. As far as is known, SOCE practitioners do not use aversive techniques and require written informed consent to ensure voluntary participation [1]. Almost all operate in religious settings and publicly denounce conversion therapy. Like conversion therapy, they are open to change in sexual orientation, but like gay-affirming therapy (GAT), they are also open to supporting persons who cannot or do not wish to change.

Despite the common view of the alternative, population research has repeatedly found SOCE to be generally safe and effective. Claims to the contrary universally rely on individual anecdotes, which are notoriously unreliable, or on LGBT (lesbian, gay, bisexual, or transgender) samples, that is, samples that exclude, by design, anyone who may have changed sexual identity. The findings of the 2009 APA task force on Appropriate Therapeutic Responses to Sexual Orientation are often mis-cited as concluding that SOCE is harmful and ineffective [2]. What the APA report actually determined was that “we cannot draw a conclusion” whether or not recent forms of SOCE were effective (p. 83) nor “how likely it is that harm will occur from sexual orientation change efforts (SOCE)” (p. 42). Population evidence since that time alleging to show harm from SOCE has been fundamentally flawed. Clinical samples before and after 2009, both cross-sectional and longitudinal, have found no evidence of undue harm and positive evidence of change, albeit limited, in sexual orientation. Spitzer, from a sample very similar to that used in the present study, reported that about two-thirds of SOCE participants experienced an incremental movement toward heterosexuality in one or more dimensions of sexual orientation, with a small minority undergoing a more or less complete heterosexual shift [3]. Jones and Yarhouse, based on repeated follow-up assessments over seven years, likewise found that just over half of 63 SOCE participants experienced incremental increases in heterosexual attractions, romantic ideation, and fantasies [4,5]. Karten and Wade found that a group of male SOCE participants reported an average decline in homosexual feelings and an increase in heterosexual feelings, with positive psychological effects [6]. Sullins reported no evidence of harm from SOCE in a U.S. population sample of LGBT persons [7]. Sullins et al., examining an earlier version of the same data as the present study, found that a majority of men undergoing SOCE reported incremental change toward heterosexuality in attractions, identity, and behavior, with a small minority attaining complete heterosexual change, in results very similar to those of Spitzer [3,8]. 

The present study attempts to move beyond demonstrations of overall change to examine more detailed questions about the type and character of change. Participants typically characterize their motivation for undergoing SOCE as distress over the presence of undesired same-sex attraction, not the absence of desired opposite-sex attraction (Note: In this paper, which draws on sources and instruments that use both sets of terms, the distinction between “homosexual” and “heterosexual” will be used interchangeably with the distinction between “same sex” and “opposite sex.”) Two questions are, therefore, of interest. First, given that heterosexual attraction and homosexual attraction may be theoretically and practically distinct, what is the relation, if any, between changes in these two types of attraction? This question will be operationalized by examining whether the change toward heterosexuality consisted more in the reduction of same-sex attraction or in the increase of opposite-sex attraction. My hypothesis is that the change toward heterosexuality consisted primarily of the reduction of same-sex attraction (H1). Second, what is the relation between a change in attraction and a change in behavior? This question will be addressed by examining whether a change in attraction toward heterosexuality resulted more in a reduction of homosexual behavior or in an increase in heterosexual behavior. My hypothesis is that the change toward heterosexuality resulted primarily in reduced homosexual sex behavior rather than in an increase in heterosexual sex behavior.

## Materials and methods

Participants

The present study analyzes an online survey of men who had engaged in a variety of SOCEs to alleviate undesired same-sex attraction. The data were originally collected for a doctoral dissertation at Southern California Seminary, which includes a full description of the survey methods, administration, and question-wording, and is archived online, with the raw data file and codebook [9]. An earlier analysis of the same data was retracted for reasons unrelated to data quality or ethical misconduct, followed by a protest from one of the co-authors [10-12]. This study addresses new questions beyond the analysis of a previous study, which contains a thorough description of the features of the data, measures, and some of the summary outcomes of these data [8]. For the sake of brevity, the present study will adapt some of the former material and language of the previous study that is pertinent to this one and will refer readers interested in more detail to the former study. 

The original survey was collected from therapy networks and religious organizations. There were a total of 158 responses, 125 of which were from men in the United States. Of these, 53 (42%) were still engaged in ongoing SOCE (“continuers”) and 72 (58%) had completed SOCE (“completers”). To reduce extraneous variation and ensure the full effect of completed SOCE on the questions of interest, the 53 continuers were dropped from the analysis.

This study engages in secondary analysis of de-identified publicly available data, a use which has been determined by the Office of Human Protection of the Department of Health and Human Services to be exempt from human subject ethical review under 45 CFR 46.104 (2018 revision). The original data collection included written informed consent and was approved by the Institutional Review Board of Southern California Seminary [9]. Analyses were performed in Stata 18. 

Measures

The original questionnaire included 77 items on issues related to SOCE exposure to assess “if the participants’ same-sex attractions, thoughts and actions were diminished or changed to thoughts, feelings, and behaviors towards the opposite sex,” and “if there were any helpful (or harmful) effects experienced due to therapy” [13]. Respondents were asked to indicate both at “six months before getting help” and “currently” how often they: 1) had homosexual sex; 2) looked with lust or daydreamed about having homosexual sex; 3) desired romantic, emotional, homosexual intimacy; 4) engaged in passionate homosexual kissing; 5) had heterosexual sex; 6) looked with lust or daydreamed about having heterosexual sex; 7) desired romantic, emotional, heterosexual intimacy; or 8) engaged in passionate heterosexual kissing. For these six items “sex” was defined as “touching genitals, oral, anal, or vaginal intercourse.” Response options, coded 1-5 for analysis, were “almost never,” “yearly,” “monthly,” “weekly,” and “almost daily" [13]. The median time post-SOCE was approximately three years.

Respondents also rated their sexual attraction both currently and six months before getting help using a modified Kinsey scale labeled “heterosexual,” “almost entirely heterosexual,” “more heterosexual than homosexual,” “bi-sexual,” “more homosexual than heterosexual,” “almost entirely homosexual,” and “homosexual” [14]. These are conventionally numbered from 0 to 6, with 0-2 indicating increasingly exclusive heterosexual attraction and 4-6 indicating increasingly exclusive homosexual attraction. For technical reasons, the responses were coded 1-7 for analysis in the present study.

Respondents also reported background demographic information including age, sex, ethnicity, income, education, marriage and children, and religiosity. These measures are discussed in detail in the former study [8].

Analyses

The goal of the analysis was to isolate groups with distinct trajectories of change in sexual attraction in order to then observe the related dimensions and components of change for these groups. This proceeded in three steps. First, mean change before and after SOCE was assessed for sexual attraction, identification, and two measures of behavior: reduction in homosexual sex and increase in heterosexual sex. Assessments were made using the Wilcoxon signed-rank test, a nonparametric test suitable for small ordinal distributions, which is evaluated like a t-test. The critical value for significance was 0.05. T-test results were very similar and would have changed only one significant conclusion that was close to the critical value.

The corresponding effect sizes (Cohen’s d) assumed a normal distribution. Effect sizes express the average change or difference in standard deviation, and thus provide a standardized indication of the magnitude of change or contrast, which is comparable across differently-scaled variables. The substantive interpretation of effect sizes is a matter of some disagreement and varies according to the variables being considered; however, an effect size below 0.2 is generally interpreted as small, 0.3-0.6 moderate, and above 0.8 as indicating a large effect.

In the second step, the Kinsey scale of sexual attraction before and after SOCE was crosstabulated to form a mobility table that revealed groups whose sexual attractions had changed toward increased heterosexual attraction, changed toward increased homosexual attraction, or had not changed. Homosexual and heterosexual versions of four measures, fantasy, desire for intimacy, kissing, and sex relations, were compared for these three groups to determine the relation between homosexual reduction and heterosexual increase (hypothesis H1). Third, a similar analysis was then performed on more focused subgroups of attraction change to examine the question regarding homosexual and heterosexual sex behavior (hypothesis H2).

## Results

Sample characteristics

As Table 1 shows, the sample participants were more highly educated, white, affluent, Western, and Mormon than the U.S. average. Over nine out of 10 respondents (91%, N=64) were white. Over seven out of 10 (73%, N=55), or about twice the proportion of all Americans, had obtained a Bachelor’s degree or higher education. About 58% (N=43) reported a household income above the 2011 national median income of $49,445. These characteristics are similar to other samples of SOCE participants in the United States [15].

**Table 1:** Sample characteristics (N=72) The data are represented in percentages. N, number of cases; AA, associate of arts; LDS, Latter-Day Saints

Variable		Percent
Total married		N=35 (49%)
	1-5 years	22.9%
	6-10	11.4%
	11-25	31.4%
	26-50	34.3%
Age		
	18-25 years	11.1%
	26-35	27.8%
	36-45	16.7%
	46-55	26.4%
	56-65	16.7%
	66+	1.4%
Ethnicity		
	African American/Black	2.8%
	Asian/Pacific Islander	1.4%
	Caucasian/White	90.1%
	Hispanic	4.2%
	Multi-racial	1.4%
Household income		
	$0-10,000	2.8%
	$10,001-$25,000	18.1%
	$25,001-$50,000	19.4%
	$50,001-$75,000	12.5%
	$75,001-$100,000	16.7%
	$100,001-$150,000	16.7%
	$150,000+	13.9%
Highest education		
	High school	1.4%
	Some college or AA degree	21.1%
	Bachelors degree	33.8%
	Masters degree	35.2%
	Doctoral degree	8.5%
Church attendance		
	Daily	1.4%
	Few times a week	23.6%
	Once a week	58.3%
	A few times a month	11.1%
	Major holidays	2.8%
	Rarely or never	2.8%
Religious affiliation		
	Unspecified Christian	35.7%
	Mormon (LDS)	31.4%
	Non-denominational Christian	15.7%
	Jewish	5.7%
	Roman Catholic	5.7%
	Baptist	2.9%
	Episcopalian	1.4%
	Methodist	1.4%
Region of residence		
	West	55.7%
	Central	11.5%
	South	11.5%
	East	21.3%

The members of the sample reported notably high religious observance. Over eight in 10 (83%, N=60) reported attending religious services weekly or more often. A quarter of respondents (25%, N=18) reported attending church more than once a week; only two respondents (3%) reported never or rarely attending religious services. By contrast, in a recent population sample of LGB-identified sexual minorities, only 9% reported at least weekly religious service attendance and 69% reported attending seldom or never [16]. The current sample members are far more religiously active and the LGB-identified sample members are far less religiously active than are Americans in general, of whom 33% reported attending religious services at least weekly and 31% seldom or never in 2016 [17]. 

Observance of religious norms of marriage and natality was also very high. Almost half (49%, N=35) of respondents were married, a proportion twice that of most samples of sexual minority persons. Thirty-one of the 35 married respondents (89%) had children, averaging 2.6 children each, or about one child higher than the U.S. average.

Almost a third of respondents (31%, N=22) identified as “Mormon” or “LDS” (Latter-Day Saints), a religious group, which comprises less than 2% of the U.S. population. The proportion of Mormons in the sample was probably even larger, since “Mormon” or “LDS” was not provided as a response option, so these were all write-in responses and an additional 35% of respondents checked “Unspecified Christian.” In addition, over half of the respondents (55%, N=40) lived in the Western region of the United States, which has a higher concentration of Mormons. The next most common religious group was “Non-denominational Christian” at 17% (N=12), followed by Roman Catholic, Baptist, Methodist, and Episcopalian. About 6% (N=4) of the sample identified as Jewish, which is over twice the concentration of Jews in the general population.

Participants reported seeking a variety of help for their sexuality-related distress, including religious peer support groups (86.1%, N=62), pastoral counselors (70.8%, N=51), marriage or family counselors (65.3%, N=47), same-sex retreats (58.6%, N=42), psychologists (54.2%, N=39), non-religious support groups (48.6%, N=35), psychiatrists (29.2%, N=21), and social workers (26.4%, N=19). Most participants utilized more than one of these.

Completers and continuers

As already noted, since the outcome of SOCE for those who were still in process was unknown, the analysis of change components was restricted to the 72 completers (58%), who indicated that they were no longer undergoing SOCE, ignoring the 53 continuers (42%), who were still involved in ongoing SOCE. As Table 2 reports, the two groups were different on change metrics in ways that suggested support for this strategy. While both groups had changed by almost identical amounts, on average, the completers differed in several respects from the continuers. First, on every metric the completers both started and finished at a lower homosexual mean than did the continuers and were slightly more likely both to have undergone a full heterosexual shift and to have changed in a homosexual direction. Four completers (5.6%) but only one continuer (2%) had changed attraction in a homosexual direction.

**Table 2:** Change toward heterosexuality in attraction, identification, and behavior following SOCE by ongoing SOCE participation (n=52) or completed SOCE (n=72) Columns labeled "Yes" denote continuers. Columns labeled "No" denote completers.  Column percentages may not total exactly 100 due to rounding. This table repeats some material from Sullins et al. (2021), Table 3.  The data are presented in percentages with standard error in parentheses.  P less than 0.05 is considered significant. SE, standard error; P, the p-value for the Wilcoxon signed-rank test for the difference of two paired ordinal distributions; SOCE, sexual orientation change efforts

		Attraction			Identification			Behavior (homosexual sex)			Behavior (heterosexual sex)		
Still currently in therapy		All	Yes	No	All	Yes	No	All	Yes	No	All	Yes	No
Mean change		% (SE)	% (SE)	% (SE)	% (SE)	% (SE)	% (SE)	% (SE)	% (SE)	% (SE)	% (SE)	% (SE)	% (SE)
	Mean before SOCE	5.7	5.9	5.6	4.8	5.1	4.6	2.4	2.5	2.3	1.7	1.7	1.6
	Mean after SOCE	4.1	4.3	4.0	3.6	3.8	3.4	1.5	1.6	1.4	2.0	1.7	2.1
	Mean change	-1.6	-1.6	-1.6	-1.2	-1.3	-1.2	-0.9	-0.9	-0.9	+0.3	0.0	+0.5
	P: difference test (Wilcoxon)	0.000	0.000	0.000	0.000	0.000	0.000	0.000	0.000	0.000	0.008	0.844	0.007
Change vectors													
Homosexual change		4.0 (1.8)	2.0 (2.0)	5.6 (2.7)	9.6 (2.6)	9.6 (4.1)	9.7 (3.5)	8.1 (2.5)	7.7 (3.7)	8.6 (3.4)	6.8 (2.2)	3.8 (2.7)	9.7 (3.5)
No change		27.4 (4.0)	33.3 (6.7)	22.2 (4.9)	36.0 (4.3)	30.8 (6.5)	38.9 (5.8)	47.2 (4.5)	46.2 (7.0)	47.1 (6.0)	75.2 (3.7)	88.5 (4.4)	62.5 (5.7)
Partial heterosexual change		50.0 (4.5)	47.1 (7.0)	52.8 (5.9)	35.2 (4.3)	40.4 (6.8)	31.9 (5.5)	8.1 (2.5)	13.5 (4.7)	4.3 (2.4)	7.5 (2.3)	1.9 (1.9)	12.5 (3.9)
Full heterosexual change		18.5 (3.5)	17.6 (5.3)	19.4 (4.7)	19.2 (3.5)	19.2 (5.5)	19.4 (4.7)	36.6 (4.3)	32.7 (6.5)	40.0 (5.9)	10.5 (2.7)	5.8 (3.2)	15.3 (4.2)

On the question of increasing heterosexual sex, the completers and continuers were notably different. The completers increased the average frequency of heterosexual sex by 0.5 (on a 5-point ordinal measure), while for the continuers the frequency of heterosexual sex did not change over the course of SOCE. While over half (54%, N=28) of continuers reported a reduction in the frequency of homosexual sex, 89% (N=46) of them reported no change in the frequency of heterosexual sex since beginning SOCE. This suggests that on this question also it is important to look at the presumably completed change in those men who reported that their SOCE participation was in the past.

Table 3 presents reported sexual attraction on the Kinsey scale before and after completing SOCE. Mean sexual attraction was 1.6 units lower after SOCE than before SOCE, indicating an average move from homosexual attraction toward greater heterosexual attraction. Change was monotonic. From before to after SOCE, Kinsey categories 1-4, at the heterosexual end of the scale, each gained cases, while categories 5-7, at the homosexual end of the scale, each lost cases. However, the decline at the homosexual end (categories 6 and 7) was twice as prevalent as the increase at the heterosexual end (categories 1 and 2). In this distribution, the movement away from strong homosexual attraction was larger than the movement toward strong heterosexual attraction.

**Table 3:** Kinsey scale of sexual attraction before and after completing SOCE (N=72) Except for the line labeled “Mean (SE),” the data are presented as case counts, with percentages in parentheses.  P less than 0.05 is considered significant. N, number of cases; SOCE, sexual orientation change efforts; SE, standard error; P, the p-value for the Wilcoxon signed-rank test for the difference of two paired ordinal distributions

		Before SOCE	After SOCE	Difference after - before	Difference test	Effect size
Kinsey scale categories		N (percent)	N (percent)	-	P	Cohen’s d
	Mean (SE)	5.6 (0.14)	4.0 (0.21)	-1.6	0.000	1.07
1	Heterosexual	0 (0)	8 (11.1)	+8	-	-
2	Almost entirely heterosexual	1 (1.4)	7 (9.7)	+6	-	-
3	More heterosexual than homosexual	4 (5.6)	16 (22.2)	+12	-	-
4	Bisexual	4 (5.6)	9 (12.5)	+5	-	-
5	More homosexual than heterosexual	21 (29.2)	18 (25.0)	-3	-	-
6	Almost entirely homosexual	24 (33.3)	6 (8.3)	-18	-	-
7	Exclusively homosexual	18 (25.0)	8 (11.1)	-10	-	-
	Total	72	72	-	-	-

**Figure 1:**
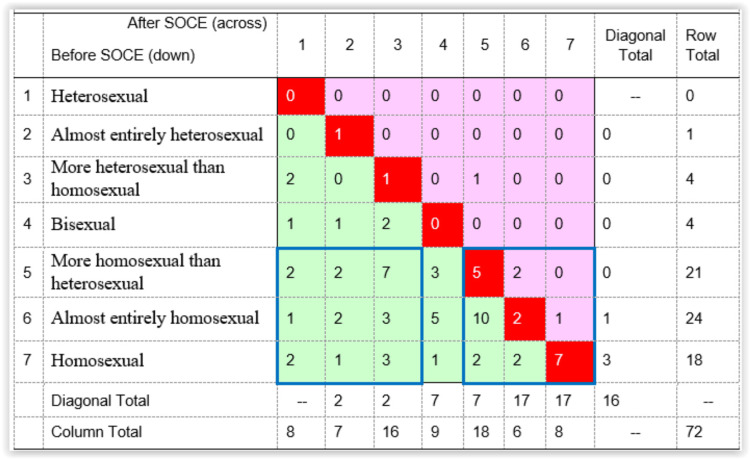
Mobility diagram of sexual attraction before and after completing SOCE (N=72) The data are presented as case counts. Cells in bright red (the diagonal) signify no change; cells in pink (upper off-diagonal) signify change toward greater homosexual attraction; cells in green (lower off-diagonal) signify change toward greater heterosexual attraction. N, number of cases; SOCE, sexual orientation change efforts

Figure 1 crosstabulates reported sexual attraction before and after SOCE, creating a mobility table diagram, which makes it easier to inspect how individual self-reported attraction changed following SOCE. The cells in the top-left to lower-right diagonal (shaded red in the figure) represent cases where sexual attraction following SOCE was the same as it was before SOCE. For these men, sexual attraction did not change over the course of SOCE. The off-diagonal cells in the remainder of the table represent cases where reported sexual attraction after SOCE was different than before SOCE. The cells to the upper right of the diagonal (shaded pink) show cases where before-to-after sexual attraction moved in a homosexual direction, whereas those in the lower left off-diagonal (shaded green) show cases where sexual attraction moved in a heterosexual direction.

From Figure 1, it is clear that 16 men (22%) reported no change in sexual attraction; four (6%) shifted toward greater homosexual attraction; and 52 (72%) shifted toward greater heterosexual attraction. This latter group corresponds to the sum of the “partial heterosexual change” and “full heterosexual change” categories of sexual attraction shown in Table 2. Table 4 compares those experiencing a heterosexual change to the two smaller groups combined, that is, men who experienced either no change or change toward homosexuality, on measures of attraction and behavior. The overall results, shown in the left panel, are a composite of the disparate outcomes of the heterosexual and the none/reverse change groups that both obscure the presence of stability and homosexual change, on the one hand, and understate the extent of heterosexual change for those who made a heterosexual shift, on the other hand. Not surprisingly, one in five men who experienced no change or change toward homosexuality, shown in the right panel, reported small, non-significant changes in every measure of sexual attraction and behavior shown in Table 4.

**Table 4:** Change in fantasies, desires, and sexual behavior, comparing those moving toward heterosexuality (lower off-diagonal of Figure 1; N=52) with the remainder (N=20) The data are represented as means with standard error in parentheses.  P less than 0.05 is considered significant. Cohen's d reports the absolute value. N, number of cases; SE, standard error; P, the p-value for the Wilcoxon test for the difference of two ordinal distributions

Dimension	All (72)				Moved toward heterosexuality (52)					No change/moved toward homosexuality (20)			
	Before	After	Difference	Test	Before	After	Difference	Test	Effect size	Before	After	Difference	Test
	Mean (SE)	Mean (SE)	B - A	P	Mean (SE)	Mean (SE)	B – A	P	Cohen’s d	Mean (SE)	Mean (SE)	B - A	P
Sexual attraction	5.63 (0.14)	4.00 (0.21)	-1.63	0.000	5.67 (0.14)	3.33 (0.20)	-2.34	0.000	1.87	5.50 (0.34)	5.75 (0.32)	+0.25	0.056
Homosexual fantasy	4.43 (0.12)	3.06 (0.17)	-1.37	0.000	4.54 (0.14)	2.78 (0.20)	-1.76	0.000	1.42	4.15 (0.22)	3.75 (0.24)	-0.40	0.149
Desire for homosexual intimacy	3.85 (0.18)	2.76 (0.18)	-1.09	0.000	3.84 (0.22)	2.19 (0.17)	-1.68	0.000	1.16	3.85 (0.32)	4.25 (0.23)	+0.40	0.214
Homosexual kissing	1.67 (0.14)	1.33 (0.11)	-0.34	0.032	1.65 (0.16)	1.0 (0)	-0.65	0.000	0.81	1.7 (0.27)	2.15 (0.32)	+0.45	0.188
Homosexual sex	2.34 (0.18)	1.41 (0.11)	-0.93	0.000	2.47 (0.23)	1.13 (0.09)	-1.34	0.000	1.08	2.00 (0.28)	2.10 (0.28)	+0.10	0.741
Heterosexual fantasy	1.94 (0.14)	3.01 (0.16)	+1.07	0.000	1.96 (0.16)	3.41 (0.17)	+1.45	0.000	1.25	1.90 (0.31)	2.00 (0.32)	+0.10	0.630
Desire for heterosexual intimacy	2.63 (0.18)	3.72 (0.18)	+1.09	0.000	2.84 (0.22)	4.22 (0.15)	+1.38	0.000	1.04	2.05 (0.32)	2.45 (0.37)	+0.40	0.135
Heterosexual kissing	1.89 (0.15)	2.41 (0.18)	+0.52	0.006	1.98 (0.17)	2.67 (0.21)	+0.69	0.005	0.49	1.63 (0.28)	1.75 (0.30)	+0.12	0.938
Heterosexual sex	1.65 (0.13)	2.11 (0.17)	+0.28	0.007	1.69 (0.15)	2.27 (0.20)	+0.58	0.012	0.46	1.55 (0.26)	1.70 (0.29)	+0.15	0.186

Isolating this group of confounders from the men who shifted toward heterosexuality, shown in the middle panel, reveals a before/after decline in Kinsey scale ranking of 2.3 in the latter group, corresponding to an effect size of 1.87, values greater than the apparent overall decline of 1.6 and effect size of 1.07 reported in Table 2. For the men reporting a change toward heterosexuality, the components of sexual attraction (fantasy, intimacy, kissing, and sex relations) after SOCE were all reduced for homosexual attraction and increased for heterosexual attraction, with a larger magnitude than that observed for the overall group. The effect sizes for homosexual change were all larger than the corresponding effect sizes for heterosexual change, though the differences in effect size are not statistically significant. For all but one measure (kissing), the raw differences are larger as well. The decline in the frequency of homosexual sex (effect size: 1.08) was more than twice as large as the increase in the frequency of heterosexual sex (effect size: 0.46). These results suggest, but cannot strongly support, a positive conclusion to the question of whether the reduction in homosexual attraction was generally larger than the increase in heterosexual attraction.

To further clarify the evidence on this question, the comparative groups reporting change or no change were specified further. Referring to Figure 1, it can be seen that of the 63 men who began in one of the homosexual categories of attraction, that is, in categories 5, 6, and 7, two distinct outcome groups can be identified. Each group consists of nine outcome cells indicated by blue borders in Figure 1. The 31 men who began in categories 5-7 and ended in those same categories after SOCE comprise men with more or less stable homosexual attraction, which was reported as generally the same after SOCE as before SOCE. By contrast, the 23 men who began in the homosexual categories 5-7 but reported sexual attraction after SOCE in the heterosexual categories 1-3 comprise men who underwent a transition from primarily homosexual to primarily heterosexual attraction.

Table 5 compares these two groups on the same measures of sexual attraction and behavior as presented in Table 4. In both groups (stable homosexual and heterosexual transition), homosexual measures dropped and heterosexual measures increased, but they did so much more strongly and clearly in the heterosexual transition group. The effect sizes for these changes were large and significant for the heterosexual transition group, whereas they were much smaller, and six of the eight were not statistically significant, for the stable homosexual group.

**Table 5:** Attraction and behavior change, stable homosexual (N=31) versus heterosexual transition (N=23) The data are represented as mean values. P less than 0.05 is considered significant. P, p-value for the Wilcoxon test for the difference of two ordinal distributions; ns, not statistically significant

	Stable homosexual (N=31)		Difference test	Effect size	Heterosexual transition (N=23)		Difference test	Effect size
	Mean before	Mean after	P	Cohen’s d	Mean before	Mean after	P	Cohen’s d
Homosexual ideation (fantasy)	4.52	3.90	.002	0.73	4.65	2.13	.000	2.28
Heterosexual ideation (fantasy)	1.48	2.03	.029	0.48, ns	2.00	3.83	.000	1.84
Homosexual intimacy	4.12	3.77	.081	0.29, ns	3.74	1.61	.000	1.52
Heterosexual intimacy	1.80	2.71	.002	0.67	3.00	4.30	.000	1.00
Homosexual kissing	1.58	1.65	.841	0.05, ns	1.96	1.00	.004	1.04
Heterosexual kissing	1.52	2.00	.060	0.36, ns	2.00	2.48	.184	0.36, ns
Homosexual sex	2.00	1.71	.360	0.22, ns	2.86	1.00	.000	1.54
Heterosexual sex	1.39	1.74	.188	0.32, ns	1.70	2.17	.134	0.38, ns

Rejecting homosexual sex, not increasing heterosexual sex

Were the reductions in homosexual measures greater than the increases in heterosexual measures? Table 6 addresses this question. For the stable homosexual group, none of the homosexual reductions were greater than the heterosexual increases. Two of the differences (ideation and sex relations) were so small as not to be significantly different from zero. The other two differences (intimacy and kissing) were at or approaching statistical significance, but for both the heterosexual differences were larger than the homosexual ones. These results are consistent with the fact that none of these men reported a definitive transition to heterosexual attraction.

**Table 6:** Homosexual reduction and heterosexual increase compared, showing before-after mean difference, stable homosexual (N=31) versus heterosexual transition (N=23) The data are represented as the difference in mean values. P less than 0.05 is considered significant. N, number of cases; P, p-value for the Wilcoxon signed-rank test for the difference of two ordinal distributions

	Stable homosexual (N=31)				Heterosexual transition (N=23)			
Change from before to after SOCE in …	Homosexual	Heterosexual	Difference in absolute values	P	Homosexual	Heterosexual	Difference in absolute values	P
Ideation (fantasy)	-0.62	+0.55	0.12	0.659	-2.52	+1.83	0.70	0.071
Intimacy	-0.33	+0.83	0.50	0.045	-2.13	+1.30	0.83	0.047
Kissing	+0.06	+0.48	0.42	0.058	-0.96	+0.48	0.48	0.495
Sex relations	-0.29	+0.35	0.07	0.608	-1.86	+0.50	1.36	0.029

By contrast, the men who did report a heterosexual transition also reported a greater homosexual decline than the heterosexual increase on all measures (though not all were statistically significant). Despite the differences in attraction change, however, neither the stable homosexual men nor the heterosexual transition group substantially increased the frequency of heterosexual sex. As Table 5 shows, the men in the stable homosexual group reported a nominal increase in average frequency, from 1.4 to 1.7 (+.3). Despite much larger changes in sexual attraction, however, the men in the Heterosexual transition group reported only a slightly larger increase, from 1.7 to 2.2 (+.5). Neither effect size was statistically significant.

In sum, the change in the frequency of heterosexual sex did not significantly differ between these two groups and did not materially increase for either one. By contrast, the changes in homosexual kissing and sex were notably different for the two groups. As Table 5 reports, for those undergoing heterosexual transition, the heterosexual effect sizes for both of these behaviors are not only smaller than the comparable homosexual effect sizes, they are not different than zero. By contrast, the comparable homosexual decrease in these behaviors is extreme: it is as large as possible.

Table 7 illustrates this point. The table presents the tabulated distributions of both behaviors before and after SOCE. In both cases, it can be seen that after SOCE, a range of frequencies from “almost never” to “almost daily” was reduced to a single response from all men reporting a heterosexual transition: “almost never.” Strikingly, all 23 men in the heterosexual transition group reported almost never engaging in homosexual kissing or homosexual sex after SOCE. Ideation and intimacy are direct measures of elements of attraction while kissing and sex are both measures of behavior. This notable unanimity on the most extreme reduction possible for both behavioral variables, in a context where the reported reduction in homosexual attraction and increase in heterosexual behavior was much less definitive, indicates a concerted effort to avoid homosexual behavior. For the men in the heterosexual transition group, undergoing SOCE was associated with some reduction in homosexual attraction, a smaller increase in heterosexual attraction, little or no increase in heterosexual sex behaviors but a definitive rejection of homosexual sex behaviors.

**Table 7:** Change in homosexual kissing and sex relations, illustrating effects from Table 6, heterosexual transition (N=23) The data are represented as case counts with percent in parentheses. P less than 0.05 is considered significant. N, number of cases; %, percent; P, p-value for the Wilcoxon test

		Heterosexual transition (N=23)		Difference test	Effect size
		Before	After		
		Count (%)	Count (%)	P	Cohen’s d
Homosexual kissing					
	Almost never	14 (36.4%)	23 (100%)	0.004	1.04
	Yearly to weekly	8 (36.4%)	0.0 (0.0%)	-	-
	Almost daily	1 (27.3%)	0.0 (0.0%)	-	-
Homosexual sex					
	Almost never	8 (36.4%)	23 (100%)	0.000	1.54
	Yearly to weekly	8 (36.4%)	0.0 (0.0%)	-	-
	Almost daily	6 (27.3%)	0.0 (0.0%)	-	-

## Discussion

This examination of sexual orientation change measures for 72 men who had completed SOCE found support for both hypotheses proposing that change consisted primarily of a reduction in homosexuality as opposed to an increase in heterosexuality. In support of H1, which states that attraction change toward heterosexuality will consist primarily in reduced same-sex attraction, for both sexual fantasy and desire for intimacy, heterosexual attraction increased significantly less than homosexual attraction decreased. The same was true even more strongly for H2, which posits that changes in behavior will also consist primarily of reduced homosexual behavior rather than increased heterosexual behavior. Men who had made a heterosexual transition not only increased the frequency of heterosexual sex less than they reduced the frequency of homosexual sex, but they did not increase it at all. At the same time, notably, they reduced the frequency of homosexual sex to the greatest extent possible.

Denial of change

The finding that some self-reported sexual orientation change did occur is consistent with previous research and with evidence that sexual orientation is not an immutable genetic trait, spontaneously changes for many over the life course, and is reported to accompany some religious change [3-6,18-22]. Notwithstanding this evidence, self-report data on homosexual to heterosexual change like the present is widely rejected on the speculation that persons undergoing SOCE “may be especially susceptible to believing and reporting that therapy has succeeded regardless of its true effectiveness” [23]. In all cases that I know, this suspicion is offered with no supporting evidence and is noticeably absent when considering similar self-reports of heterosexual to homosexual change (“coming out”). Proponents of this argument ignore the fact that samples that include only LGB-identified respondents almost universally report an absence of change from SOCE, whereas samples in which respondents are not screened for LGB identity, like the present one, generally report modest, mostly partial, change in sexual orientation. To my knowledge, none have offered any rationale why those who report an extreme lack of change should be believed but those who report moderate change should not be believed. Because this bias has been used not only to challenge but also to censor the publication of evidence such as the present data, and even to criminalize the kind of therapy it examines, the issue merits extended treatment in this section. 

Evidence of sexual orientation change challenges the claim that sexual orientation is an immutable feature of personal identity, which has become an axiom of the legal and political construction of LGBT identity. Proponents of this view claim that, for LGBT-identified persons, the possession of same-sex attraction or its cognate is “who they are.” Any attempt to change their unchangeable sense of sexual attraction (or gender identity) threatens their ontological personhood, causing substantial psychological distress, and will inevitably fail.

On this view, efforts to change same-sex sexual orientation or its cognate are inherently unethical and should be banned. Evidence that persons have successfully changed same-sex sexual orientation and/or have attempted to do so without undue psychological harm is considered even more unethical and should be censored lest it harm LGBT persons. For some scholars, this is especially the case if such evidence is credibly true [24]. Because immutability is a universal claim and grounds ontological arguments, evidence that even a few persons have credibly undergone homosexual to heterosexual change can threaten the legal and political construction of LGBT identity [25]. For such reasons, evidence of change such as that presented in this study has been aggressively denounced and even retracted on dubious grounds in some scientific settings [12,26]. 

A group of prominent psychologists on this issue disclosed that they “strongly doubt” that “sexual orientation can be changed with therapy,” and therefore are suspicious of self-reported evidence of change, because of physiological research showing that male physiological arousal patterns stimulated by images of the same sex cannot be changed to similar arousal patterns prompted by images of the opposite sex [23]. Indeed, if male sexual orientation change is defined as a change in physiological arousal from exclusively male to exclusively female sex object stimulation, then the evidence presented in this study is consistent with the claim that none of the SOCE completers changed sexual orientation. As Table 4 shows, only two participants (3%) reported moving over the course of SOCE from Kinsey category 7 (what Kinsey termed “exclusively homosexual”) to Kinsey category 1 (“exclusively heterosexual”). It is certainly possible that these two respondents may have misunderstood the question or misreported the extent of their change in attraction.

This concern cannot apply to the sample at large, however, as almost none of them reported exclusive sexual attraction either homosexual or heterosexual. This feature of the data confirms Savin-Williams' contention that the construction of sexual orientation assumed by Bailey et al. imposes a false binary dichotomy, either only homosexual or only heterosexual, on a wide range of possibilities, most if not all of whom lay between or outside these two alternatives [23,27]. Table 3 even suggests some regression to the mean, a feature of continuous distributions. Apart from the two respondents just noted, the remaining 97% (N=70) of the sample began and/or ended with some level of mixed attraction to both sexes. Thus, for almost all men in this sample, any reported change in sexual orientation did not entail the kind of reversal of essential biological arousal patterns that Bailey et al. believe is not possible, even if the Kinsey spectrum of sexual attractions was accurate [23].

The almost universal report of mixed, not exclusive, sexual attraction in this sample is consistent with recent genetic research, which found that all persons have, or have the capacity for, attractions to both sexes in complex ways, not in a zero-sum range from homosexual to heterosexual such as the Kinsey scale requires [18]. It is also consistent with numerous other applications of the Kinsey scale, including by Kinsey himself, which have found that the large majority of nonheterosexual men report mixed sexual attractions between the two extremes of the scale [14,28].

The insistence that one’s sexual orientation self-understanding must be subordinate to this attraction binary, further, is not consistent with the well-known experience and construction of sexual minority identity in other areas. For example, a sizable proportion of children enter same-sex families by means of a parent who was previously in an opposite-sex sexual relationship [29]. Most such same-sex parents identify as gay or lesbian, not bisexual, and many are men. Clearly, it must be acknowledged that men who have transitioned from an opposite-sex to a same-sex sexual relationship have in some sense changed sexual orientation. I am not aware of a single academic publication that questions the veracity of these men or argues that they have not really changed, a fortiori to the point of advocating a criminal ban on therapy to assist them in doing so. In whatever sense it is understood and accepted that these men have transitioned from heterosexual to homosexual orientation, why is it not possible to also accept and understand that other men may transition from homosexual to heterosexual orientation?

As another example, it is widely affirmed, often by the same scholars and activists that reject reports of homosexual to heterosexual transition, that some persons come to realize that their subjective gender identity does not conform to their phenotypic birth sex. Such persons transition from one gender identity to another, while their bodily sexual biological functions can only change cosmetically. Many of the scholars and activists who reject the possibility of homosexual-to-heterosexual change in orientation in the face of intractable biological arousal patterns do not likewise reject but affirm, the possibility of male-to-female or female-to-male change in the face of intractable biological reproduction patterns. In the same sense that one person can change gender identity without changing their underlying biological sex, why is it not possible for another person to change sexual orientation without changing their underlying sexual arousal patterns?

Contrasting philosophies

At the root of the dispute over the possibility of homosexual to heterosexual orientation change lay two incommensurable philosophies of the place of sexual attraction in personal identity. For secular psychologists and gay activists, on the one hand, authentic personal identity entails the expression of sexual desire according to one’s internal or underlying orientation. On this view, to attempt to live as heterosexual when one’s internal desire is homosexual is to deny one’s true self, with attendant psychological harm.

On the other hand, the men in this sample generally hold that same-sex attraction must not be indulged but must be resisted to achieve authentic personal integration. As already noted, the participants in this study were far more religiously observant and Mormon than both the sexual minority population and Americans in general [16,17]. The religious belief systems subscribed to by most of these highly religious men present homosexual behavior as a sin, that is, impermissible behavior for religiously observant persons [30]. In this view, same-sex attraction is not understood as an authentic expression of the self, but as a temptation to behavior that impedes authentic selfhood [31]. Temptation, which is the desire to sin, is a universal feature of human imperfection, and often cannot be escaped, but human fulfillment comes from resisting temptation and avoiding sinful behavior. The extreme reduction in homosexual sex despite a degree of ongoing homosexual desire, in these very religious men following SOCE, is consistent with this understanding. 

Thus, while on the first view, psychological scholars may assert that these men have not changed sexual orientation due to possible persistent same-sex desire, on the second view these religious men may assert that they have changed sexual orientation because they are able to resist acting on any persistent same-sex desire. At play are divergent philosophies of human fulfillment, which underlie incommensurable claims about the place of sexual attraction in personal identity. The absence of exclusivity in sexual attraction for all or almost all sexual minority persons likely renders both assertions fragile and often impermanent, with many defections on both sides.

Both positions recognize that sexual orientation change understood as adopting different behaviors in the face of a less complete change in attraction may be more achievable than change understood as a change in physiological attraction alone. Bailey et al. acknowledged that in the absence of change in physiological attraction, “one can certainly make choices about whether one will or will not engage in same-sex or opposite-sex sexual behavior or become celibate. These sorts of choices likely explain claims by ex-gays and ex-lesbians that they are no longer leading a ‘homosexual lifestyle’” (p. 86) [23]. While sex psychologists concerned with physiological states may not consider such a change in behavior to constitute sexual orientation change, religious adherents concerned with holy living may perceive it to be the most important change in sexual orientation that could take place. In this case, the debate over sexual orientation change may be largely a matter of differing definitions of what constitutes change. 

Limitations

The applicability of the present study's findings is limited in several ways. Although I have argued above that the present data should not be dismissed because of undue suspicion or bias, they should nevertheless be evaluated with the same caution regarding effective recall and desirability bias that attends all self-report data. The sample of this study was not drawn in such a way as to be statistically representative of any larger population. It is not very diverse, consisting entirely of men who were disproportionately white, highly religious, and of the Mormon faith; qualities shared with a number of other samples of SOCE participants [15]. In particular, these findings should not be generalized to the general LGBT population, which do not typically share these characteristics. Marital status likely mediated the differences in sexual behavior found in this study; however, stratifying the two attraction change groups (stable homosexual and heterosexual transition) by the four marital status possibilities (continuously unmarried, continuously married, married before SOCE but not after, and married after SOCE but not before) resulted in small, single-digit design cells that were too sparse for analysis. Future research to address this question with better data would be valuable. 

## Conclusions

Reported changes in sexual orientation following completed SOCE ranged from increased homosexuality for a few men (6%, N=4), no change for about one in five (22%, N=16), generally stable homosexual attraction for over four in 10 (43%, N=31), and heterosexual transition for about a third (32%, N=23). For those undergoing a heterosexual transition, reported homosexual desire decreased more than reported heterosexual desire increased. This dynamic was even more extreme for change in sexual behavior: homosexual sex behavior dropped to the least possible amount, and heterosexual sex did not increase. This probably reflects conformity to strong religious norms against homosexual behavior but not attractions, for this group of extremely religious men. 

## Appendices

The raw data examined in this study, with codebook and questionnaire, are publicly available via the Figshare Data Repository: Replication Data for: What do sexual orientation change efforts (SOCE) change?

Raw Data File: PLS2011 data USonly.sav (Version 1) https://doi.org/10.6084/m9.figshare.26300401

Codebook: PLS2011 Codebook.rtf (Version 1) https://doi.org/10.6084/m9.figshare.26300395

Questionnaire: PLS Questionnaire 2011 (Version 1) https://doi.org/10.6084/m9.figshare.26300398.v1

Documentation: Santero P: The Effects of Therapy on U.S. Men with Unwanted Same Sex Attraction (Unpublished Psy.D. Dissertation) https://doi.org/10.6084/m9.figshare.26535706.v1
